# Development of essential oils as skin permeation enhancers: penetration enhancement effect and mechanism of action

**DOI:** 10.1080/13880209.2017.1312464

**Published:** 2017-04-12

**Authors:** Qiudong Jiang, Yeming Wu, Hui Zhang, Pei Liu, Junhong Yao, Peijun Yao, Jun Chen, Jinao Duan

**Affiliations:** a Jiangsu Collaborative Innovation Center of Chinese Medicinal Resources Industrialization, Nanjing University of Chinese Medicine, Nanjing, China;; b Pharmaceutical Research Laboratory, School of Pharmacy, Nanjing University of Chinese Medicine, Nanjing, China

**Keywords:** Transdermal, ibuprofen, chuanxiong oil, ATR-FTIR

## Abstract

**Context:** Essential oils (EOs) have shown the potential to reversibly overcome the stratum corneum (SC) barrier to enhance the skin permeation of drugs.

**Objective:** The effectiveness of turpentine, *Angelica*, chuanxiong, *Cyperus,* cinnamon, and clove oils were investigated for the capacity and mechanism to promote skin penetration of ibuprofen.

**Materials and methods:** Skin permeation studies of ibuprofen across rat abdominal skin with the presence of 3% w/v EOs were carried out; samples were withdrawn from the receptor compartment at 8, 10, 22, 24, 26, 28, 32, 36, and 48 h and analyzed for ibuprofen content by the HPLC method. The mechanisms of penetration enhancement of EOs were further evaluated by attenuated total reflection-Fourier transform infrared spectroscopy (ATR-FTIR) analysis and determination of the properties of EOs. Moreover, the toxicities of EOs on skin cells were also measured.

**Results:** The enhancement ratio (ER) values of turpentine, *Angelica*, chuanxiong, *Cyperus*, cinnamon, clove oils and azone were determined to be 2.23, 1.83, 2.60, 2.49, 2.63 and 1.97, respectively. Revealed by ATR-FTIR analysis, a linear relationship (*r* = 0.9045) was found between the ER values and the total of the shift of peak position of SC lipids. Furthermore, the results of HaCaT skin cell toxicity evaluation revealed that the natural EOs possessed relatively lower skin irritation potential.

**Conclusion:** Compared with azone, the investigated EOs possess significantly higher penetration enhancement effect and lower skin toxicity. EOs can promote the skin permeation of ibuprofen mainly by disturbing rather than extracting the SC lipids.

## Introduction

It is generally accepted that the barrier to penetration of drugs through skin is primarily located in the uppermost layer of epidermis called the stratum corneum (SC). To overcome the barrier property of SC, one of the most widely used strategies to increase percutaneous absorption would be the use of penetration enhancers (PEs) in the transdermal drug delivery systems (TDDS) (Parhi et al. 2012). PEs are the agents that ideally cause a temporary, reversible reduction in the barrier function of the SC in order to facilitate safe and effective drug delivery through the skin (Narishetty & Panchagnula 2005). Over the years, by extensive screening and testing, different classes of chemicals, such as azone, sulphoxides, pyrrolidones, alcohols, and surfactants, have been identified as PEs. Moreover, essential oils (EOs) and their volatile constituents have also been identified as one promising group of candidates to be employed as clinically acceptable PEs. Until now, EOs have been shown to be successful in delivering a range of different drugs across the skin, including vitamins (Valgimigli et al. 2012), estradiol (Monti et al. 2002), labetolol hydrochloride (Jain et al. 2008), trazodone hydrochloride (Das et al. 2006), tetramethylpyrazine (Shen et al. 2013), carvedilol (Amin et al. 2008), 5-fluorouracil (5-FU) (Lan et al. 2014a), flurbiprofen (Zhang et al. 2006; Charoo et al. 2008), diclofenac sodium (Akbari et al. 2015), indomethacin (Lan et al. 2014a) and ibuprofen (Luo et al. 2007; Shen et al. 2007; Khan et al. 2011).

EOs, derived from aromatic plants, are complex aromatic volatile mixtures of compounds having low molecular weights and diverse chemical structures. Like other PEs, EOs and their volatile constituents can enhance penetration of different drugs from topical formulation into the lower skin layers using different mechanisms of action based on (1) disintegration of the highly ordered intercellular lipid structure between corneocytes in SC, (2) interaction with intercellular protein to induce their conformational modification, (3) increase the partitioning of a drug into SC (Parhi et al. 2012; Herman & Herman 2014). Furthermore, EOs and their components can be regarded as safe PE because they are rapidly metabolized, not accumulated in the organism and fast excreted after application to the skin (Herman & Herman 2014).

Terpenes are the main constituents of EOs and consist of isoprene (C_5_H_8_) units. They have been suggested as promising nontoxic, nonirritating PEs for both hydrophilic and lipophilic drugs (Furuishi et al. 2013; Moghadam et al. 2013). Terpenes of natural origin have a ‘Generally Regarded As Safe’ (GRAS) status with the Food and Drug Administration of the United States of America (Fox et al. 2011). The penetration enhancement effectiveness of terpenes were investigated and compared. Terpenes mainly acted on the intercellular lipid structure between corneocytes to increase the fluidity of SC lipids (Furuishi et al. 2013). For the terpene families, the apparent degree of SC lipid disorder may be related to the overall size and degree of long chain alkyl functionality. Consequently, terpenes having primarily ring structures, such as menthol, have less of an effect when compared to long chain alkyl containing compounds, such as nerol (Moghadam, et al. 2013). Moreover, since they are volatile compounds, the boiling points of terpenes play a major role in their penetration enhancement efficiency (Narishetty & Panchagnula 2004). Terpenes with lower boiling points seemed to possess higher penetration enhancement efficacy. As to sesquiterpenes, the oxygenated sesquiterpenes showed a higher ability to promote drug permeation compared to hydrocarbons (Fang et al. 2003). In addition, combination of terpenes can also increase the permeation enhancement effect (Prasad et al. 2007).

The enhancement permeation capacities of the whole EO were proved to be significantly higher than its main terpene components. For example, Niaouli oil and its main terpene components (1,8-cineole, α-pinene, α-terpineol and d-limonene) were compared as PE to promote permeation of estradiol through skin (Monti et al. 2002). At the concentration of 10%, the permeation enhancement values of Niaouli oil, 1,8-cineole, α-pinene, α-terpineol, d-limonene and the mixture of terpenes were determined to be 52.1, 33.0, 8.1, 2.0, 9.4 and 46.3, respectively. Similar results were obtained in the application of EO from *Zanthoxylum bungeanum* Maxim. (Lan et al. 2014b). It was found that *Z. bungeanum* oil showed lower toxicities in skin cells compared with its main terpene components (terpinen-4-ol, 1,8-cineole and limonene) used alone. The permeation enhancement capacities with all the tested agents were in the following increasing order: terpinen-4-ol ≈ 1,8-cineole < limonene < *Z. bungeanum* oil. Due to their high permeation enhancement efficiency and low irritancy potential, increasing attention has been paid to the application of EOs in TDDS. However, in contrast to terpenes, the rule between penetration enhancement effect and mechanism of action has yet to be determined for EOs.

Ibuprofen is a classical non-steroidal anti-inflammatory drug (NSAID) which is very effective for the systemic treatment of rheumatoid arthritis, osteoarthritis, ankylosing spondylitis, and dysmenorrhea. Unfortunately, after oral administration, NSAIDs cause an increased risk of serious gastrointestinal adverse events including bleeding, ulceration, and perforation of the stomach or intestine. Ibuprofen was formulated into TDDS to reduce the side effects and avoid the hepatic first-pass metabolism. But it is difficult to maintain effective blood concentrations of ibuprofen due to its poor skin permeability (Beetge et al. 2000). To improve the percutaneous absorption, several EOs have been successfully applied to the TDDS of NSAIDs such as diclofenac sodium (Akbari et al. 2015), indomethacin (Lan et al. 2014a) and ibuprofen (Luo et al. 2007; Shen et al. 2007; Khan et al. 2011). However, to the best of our knowledge, no studies focused on the correlation between the mechanism of action and penetration enhancement effect of EOs.

The purpose of the present study was to evaluate six terpene-containing EOs including turpentine, *Angelica*, chuanxiong, *Cyperus*, cinnamon and clove oils for their capacity to promote transdermal permeation of ibuprofen. Although these EOs are traditionally used in medicine for their biological activities, little information is available from the literature on their skin permeation enhancement mechanisms.

## Materials and methods

### Materials

The herbs were purchased from the Tongling Medicinal Material Company (Tongling, China) and identified by Professor Jianwei Chen (Nanjing University of Chinese Medicine, Nanjing, China). The voucher specimens were deposited in the herbarium of Jiangsu Collaborative Innovation Center of Chinese Medicinal Resources Industrialization.

*Angelica*, chuanxiong, *Cyperus*, cinnamon, and clove oils were extracted by the steam distillation method from Radix Angelicae Sinensis [*Angelica sinensis* (Oliv.) Diels (Umbelliferae)], Rhizoma Chuanxiong [*Ligusticum chuanxiong* Hort. (Umbelliferae)], Rhizoma Cyperi [*Cyperus rotundus* L. (Sedge)], *Cinnamomum cassia* Presl. (Lauraceae), and Flos Caryophylli [*Eugenia caryophyllata* Thunb. (Myrtaceae)], respectively.

Turpentine oil was provided by Shanghai Lingfeng Chemical Reagent Co. Ltd. (Shanghai, China). Ibuprofen and 3-(4,5-dimethylthiazol-2-yl)-2,5-diphenyltetrazolium bromide (MTT) were obtained from Sigma-Aldrich Inc. (St Louis, MO). Azone was obtained from Sinopharm Chemical Reagent Co. Ltd (Shanghai, China). Acetonitrile was HPLC-grade from Merck (Darmstadt, Germany) and deionized water was purified by a Direct-Q5 super purification system (Milipore, Billerica, MA). All the other chemicals were purchased from Nanjing Chemical Reagent Corporation (Nanjing, China) and of analytical grade.

### Animals

Male Sprague-Dawley (SD) rats (200 ± 20 g) were obtained from Shanghai Jiesijie Laboratory Animal Co. Ltd (Shanghai, China) with the license number SCXK (Shanghai) 2013-0006. They were housed in Plexiglas cages at 22 ± 2 °C, relative humidity 55 ± 5% with 12 h light/dark cycle and provided with standard pellet diet with tap water *ad libitum*. Animal experiments were performed in accordance to the Principles of Laboratory Animal Care and Use in Research (Ministry of Health, Beijing, China). The protocols of animal experiments were approved by the Animals Ethics Committee of Nanjing University of Chinese Medicine.

### GC-MS analysis of the EOs

EOs were analyzed with GC-MS using an Agilent 7890 A gas chromatograph interfaced to an Agilent 5975 C inert MSD with Triple-Axis Detector (Agilent Technologies, Santa Clara, CA, United States). A NIST library was used for identifying the components. EO of 0.5 μL was injected into a HP-5 MS capillary column (30 m × 0.25 mm, 0.32 μm, i.d.) using a helium as gas carrier at 1 mL/min flow rate. Mass spectra were recorded from 30 to 650 m/z. Individual components were identified by matching their 70 eV mass spectra with those of the spectrometer database as well as by comparison of the fragmentation pattern with those reported in the literature.

### HPLC analysis of ibuprofen

The HPLC system (Shimadzu Corporation, Kyoto, Japan) for analysis was equipped with two LC-20AT pumps, a SPD-20 A UV-VIS detector and a SIL-20 A autosampler. The mobile phase consisted of acetonitrile and water (adjusted pH to 3.0 with phosphoric acid) (58:42, v/v). Separation was carried out at 25 ± 0.1 °C using a reverse-phase C18 column (Inertsil ODS-3, 5 μm, 4.6 mm × 250 mm, Hanbang Corp., Huaian, China).The detection wavelength was 220 nm and a flow rate of 1.0 mL/min was employed. A sample volume of 10 μL was injected.

### Determination of apparent partition coefficient of ibuprofen

The apparent partition coefficient of ibuprofen was measured by shake-flask method. Ibuprofen was dissolved in *n*-octanol which was presaturated with deionized water. The resulted solution was mixed with deionized water (presaturated with *n*-octanol) in equal volume, shaken, and incubated in a water bath at 37 °C for 12 h. After centrifugation, the concentrations of ibuprofen in the aqueous phase and in the octanol phase were determined by HPLC following dilution with methanol. Then the apparent partition coefficient was obtained by the ratio of the concentration in octanol phase to that in aqueous phase.

### Volatilization of EOs

The weight loss method was employed to determine the volatilization rates of EOs (Chen et al. 2014). Briefly, an excessive amount of EO was placed at 50 °C in the crucible, and the total weight was determined at predetermined time intervals. The cumulative amount of EO losing per unit area was plotted against time. The slope of the plot gave the volatilization rate. The relative density was also measured by a Sartorius BT-25 S electronic balance (Goettingen, Germany) using pure water as control.

### Solubility studies

The binary solvent mixture, propylene glycol (PG): isopropyl alcohol (IPA) (3:7, v/v) (Charoo et al. 2008), was used as a vehicle to dissolve EOs. To determine the saturation solubility of ibuprofen in vehicle, with and without 3% w/v EO, excess drug was added to known volumes of the vehicle, vortexed for 5 min followed by sonication for 10 min to dissolve the drug, and then equilibrated at 32 °C for 24 h. Finally, the saturated solution was centrifuged at 12 000 rpm for 3 min and aliquots of the supernatant were diluted with methanol and analyzed by HPLC.

### Preparation of full thickness skin and SC

After sacrificing the rats with excess ether inhalation, hair from the abdominal surface was removed with an animal hair clipper with an extreme precaution not to impair the skin. The shaved skin was then excised from the animals. The subcutaneous tissue was removed surgically and the dermal side was wiped with a cotton swab to remove the adhered fat tissue. The prepared skin was subsequently washed with normal saline, wrapped in aluminum foil, and stored at −20 °C (used within two weeks).

To obtain SC, the excised skin was immersed in 0.1% trypsin in PBS at room temperature for 10 h. The SC sheets were carefully separated from remaining layers of skin, washed thoroughly with deionized water, dried and stored in a vacuum desiccator. Finally, the SC was dipped in acetone solution for 20 s to remove sebaceous lipids and dried again.

### Measurement of SC/vehicle partition coefficient of ibuprofen

Partition of ibuprofen between the vehicle and the powdered SC was measured using the reported method (Vaddi et al. 2002; Lan et al. 2014a). The SC samples were pulverized in a mortar with a pestle. The particles that passed through 48-mesh but retained by 80-mesh sieve was used (180 ∼ 300 μm). 3% w/v EO (1 mL) dissolved in vehicle containing 0.3% w/v ibuprofen was added to 10 mg ground SC with frequent vortexing. The control was treated with vehicle containing 0.3% w/v ibuprofen. The mixture was equilibrated at 32 °C for 48 h, for the same duration as described in the permeation studies. The supernatant solution, obtained by centrifugation (12000 rpm, 10 min), was analyzed for the drug content. All partition studies were conducted in triplicate. The partition coefficient (K) of the ibuprofen was calculated using the following equation:

K = (ibuprofen concentration in SC)/(ibuprofen concentration in vehicle).

### *In vitro* skin permeation studies

The skin, rinsed with normal saline solution three times, was clamped between the donor and the receptor chamber of the Franz diffusion cell with an effective permeation area of 3.14 cm^2^ and a receiver cell volume of 8 mL. Normal saline containing 35% ethanol was applied as the receptor solution.

In permeation studies, both EO and ibuprofen were dissolved in binary solvent mixture composition of PG: IPA (3:7, v/v) to obtain 3% and 0.3% w/v concentration, respectively. The resultant solution (1 mL) was added in the donor compartment and incubated at 37 °C using a water bath with a magnetic stirrer at 500 rpm. Samples (0.5 mL) were withdrawn from the receptor chamber at predetermined time intervals (8, 10, 22, 24, 26, 28, 32, 36 and 48 h) and then replaced with an equal volume of fresh medium. The receptor fluid samples were then analyzed by HPLC for ibuprofen content.

The cumulative amount of drug permeated through a unit area of skin was plotted against time. The permeation rate (flux) was calculated from the slope of the linear portion of the plot. The cumulative amount of drugs permeating through the skin at 48 h (*Q*_48_) was calculated from the drug concentration in the receiver compartments. The lag time (*T*_lag_) was calculated by extrapolating the linear region of this plot of the curve to the X-axis. To compare the permeation enhancement capacities of each EO, the enhancement ratio (ER) was determined as follows:

ER = (flux of ibuprofen with EO)/(flux of ibuprofen without EO).

### Attenuated total reflection-fourier transform infrared spectroscopy (ATR-FTIR) studies

ATR-FTIR studies were performed in a similar manner to that reported by Furuishi et al. (2013). The skin prepared using the procedure as described above was cut into approximately 1 cm^2^ pieces and soaked in 3% w/v EO dissolved in the vehicle of PG:IPA (3:7, v/v), 3% azone or blank vehicle at 32 °C for 12 h, respectively. Then the treated skin samples were washed with distilled water and blotted dry. The infrared spectra of skin samples were obtained using FTIR spectroscopy (FTIR-230 spectrometer, JASCO Co., Tokyo, Japan) with an ATR unit (ATR-500/M, JASCO Co., Tokyo, Japan). The spectrum recorded represents an average of 32 scans obtained with a resolution of 2 cm^−1^ at room temperature. The spectra were collected in the wave number range of 4000 ∼ 650 cm^−1^. The internal reflectance element (IRE) used in this study was a zinc selenide trapezoid having 45° entrance and exit faces. Skin was carefully mounted on the IRE.

### Skin cell viability assay

HaCaT (epidermal keratinocytes) cell lines were obtained from KeyGen Biotech Co. (Nanjing, China). The cells were incubated in minimum essential medium (MEM Eagles with Earle’s Balanced Salts) supplemented with 10% heat-inactivated fetal bovine serum and 100 U/mL penicillin/streptomycin in a humidified incubator at 37 °C and 5% CO_2_.

The MTT assay was used to monitor the toxicity of EOs on human skin cells *in vitro* (Lan et al. 2014a). HaCaT cells were seeded into 96-well plates at a density of 7000 cells in a 100 μL medium per well. After 12 h, the cells were incubated with varying concentrations of EOs in a culture medium with 1% DMSO for 24 h at 37 °C. The cells that were treated with culture medium containing 1% DMSO were used as the control. Then the medium was replaced by a fresh medium containing 20 μL MTT solution (5 mg/mL in PBS) and the cells were incubated again for 4 h. Subsequently, the medium was removed and 150 μL DMSO was added to dissolve the formazan crystals. The plate was incubated for 10 min while shaking. The absorbance was measured at 490 nm using a Chromate-4300 microplate spectrophotometer (Awareness Technology Inc., Palm City, FL). Cell viability was calculated according to the following equation:

Cell viability = (A-B)/A×100%

where A was the absorbance of the control and B was the absorbance of the cells incubated with EOs or azone, respectively. All samples were evaluated in sextuplicate.

### Statistical analysis

The results were expressed as mean ± S.D. and evaluated with one-way ANOVA following by Student’s two-tailed unpaired *t*-test. A *p*-value of less than 0.05 was considered statistically significant and *p* less than 0.01 being very significant.

## Results

### GC-MS analysis of the EOs

The constituents of each EO were identified by GC-MS. The major components of *Angelica*, chuanxiong, cinnamon, clove oils were ligustilide (79.32%), ligustilide (41.00%), (*E*)-cinnamaldehyde (83.30%) and eugenol (80.22%), respectively. For turpentine oil, the main components were 1,4-cineole (26.42%), camphene (18.68%) and α-pinene (17.65%). And the main components of *Cyperus* oil were determined to be dehydrofukinone (27.41%), cyperene (27.15%) and α-cyperone (7.97%).

### Determination of apparent partition coefficient and solubility of ibuprofen

The logP value of ibuprofen between *n*-octanol and water was determined to be 2.72 ± 0.08 (*n* = 3). Lipophilic property of ibuprofen makes it an appropriate candidate for transdermal delivery (Stahl et al. 2011). It is generally accepted that the optimal log octanol/water coefficient for a drug to penetrate the SC is in the range of 1 ∼ 3 (Bartosova & Bajgar 2012).

### Volatility of EOs

The volatilization rates of turpentine, *Angelica*, chuanxiong, *Cyperus*, cinnamon and clove oils were measured to be 65.35 ± 6.06, 2.18 ± 0.32, 1.67 ± 0.09, 0.759 ± 0.028, 0.971 ± 0.025 and 0.449 ± 0.021 mg/h/cm^2^ (*n* = 3), respectively. Turpentine oil was much easily evaporated and the volatilization rate was increased about 30 ∼ 150 times compared to other EOs.

### Determination of solubility of ibuprofen

**Figure 1.**
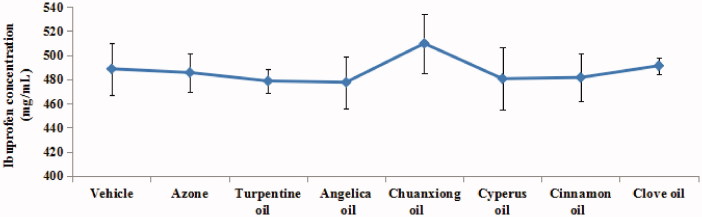
Solubility of ibuprofen with the presence of different EOs (*n* = 3). Note: vehicle = propylene glycol (PG): isopropyl alcohol (IPA) (3:7, v/v).

The saturation solubility of ibuprofen was measured to evaluate the effect of EOs on thermodynamic activities of the drug (Narishetty & Panchagnula 2005; Lan et al. 2014b). The solubility results of ibuprofen in 3% w/v EO are shown in Figure 1. There was no significant difference among the ibuprofen solubility in vehicle, 3% w/v EOs and 3% w/v azone, indicating that thermodynamic activity of drug in donor compositions used for *in vitro* permeation studies was not dramatically different. However, the solubility of Ibuprofen in 3% w/v chuanxiong oil was a little higher compared to that in other EOs.

### Measurement of SC/vehicle partition coefficient of ibuprofen

To optimize a TDDS for therapeutic efficacy generally implies that the flux of drug into the skin be maximized. Such a requirement means that the SC/vehicle partition coefficient of the drug should be enhanced as much as possible (Surber et al. 1990). As evidenced in Table 1, the presence of most EOs (turpentine, chuanxiong, *Cyperus*, cinnamon, and clove oils) led to significant (*p* < 0.05) increase of the SC/vehicle partition coefficients of ibuprofen, which indicated that these EOs might contribute to the partitioning of lipophilic ibuprofen into SC. EOs enhanced partition of the drug into SC probably by increasing the ibuprofen solubility in SC lipids (Vaddi et al. 2002). The higher solubility of ibuprofen in SC was expected to improve its skin permeation.

**Table 1.** Effect of 3% w/v EO on SC/vehicle partition coefficients of ibuprofen (*n* = 3).

EO	Partition coefficient	Ratio^a^
Vehicle	0.41 ± 0.06	1.00
Azone	0.51 ± 0.06	1.24
Turpentine oil	0.72 ± 0.01***	1.76
*Angelica* oil	0.51 ± 0.09	1.24
Chuanxiong oil	0.75 ± 0.01***	1.83
*Cyperus* oil	0.68 ± 0.09*	1.66
Cinnamon oil	0.80 ± 0.12**	1.95
Clove oil	0.98 ± 0.07***	2.39

* *p* < 0.05,

** *p* < 0.01,

*** *p* < 0.001 vs. the vehicle group.

a Ratio = (partition coefficient with 3% w/v EO treatment)/(partition coefficient with vehicle treatment).

### In vitro *skin permeation studies*

**Figure 2.**
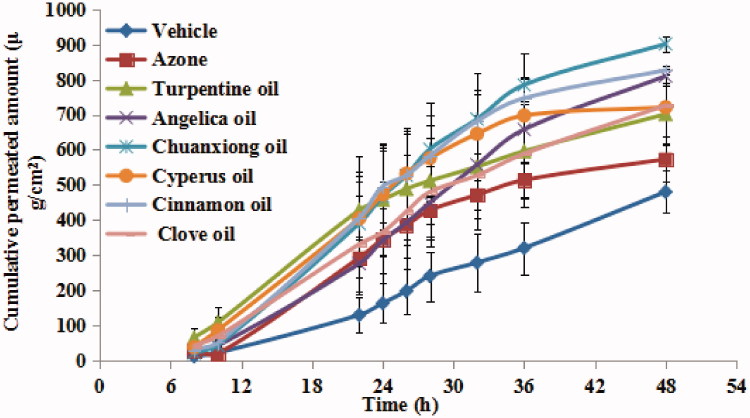
Permeation profiles of ibuprofen with 3% w/v EOs through excised rat skin (*n* = 5).

The permeation profiles of ibuprofen combined with different EOs, over a period of 48 h, are shown in Figure 2. The corresponding permeation parameters are listed in Table 2. Compared to vehicle, all EOs significantly (*p* < 0.05) enhanced the steady state flux of ibuprofen. And the cumulative permeation ratio was also significantly (*p* < 0.05) increased. Among the EOs, the highest permeation rates were observed with cinnamon and chuanxiong oils. And the results of ANOVA analysis revealed that there was significantly difference (*p* < 0.05) among different EO groups. With the presence of 3% w/v EOs, the percutaneous permeation flux of ibuprofen was increased in the following order: cinnamon oil ≈ chuanxiong oil > C*yperus* oil > turpentine oil > clove oil > *Angelica* oil. The application of cinnamon, chuanxiong, and *Cyperus* oils significantly (*p* < 0.05) increased the penetration efficiency of ibuprofen compared with azone, the well-established and standard PE.

**Table 2.** The percutaneous permeation parameters of ibuprofen with various EOs through excised rat skin (*n* = 5).

PE	Flux/μg·cm^−2^·h^−1^	ER	T_lag_/h	Permeation ratio^a^/%
Vehicle	9.94 ± 3.36	1.00	7.49 ± 0.37	47.09 ± 11.90
Azone	19.49 ± 2.66**	1.77	7.28 ± 0.93	56.22 ± 12.56
Turpentine oil	24.54 ± 7.90**	2.23	6.16 ± 0.70**	68.80 ± 17.16*
*Angelica* oil	20.22 ± 5.29**	1.83	7.57 ± 0.27	79.43 ± 5.36***
Chuanxiong oil	28.61 ± 7.66**^#^	2.60	7.94 ± 0.29	88.41 ± 4.12***^#^
*Cyperus* oil	27.37 ± 7.07**^#^	2.49	7.03 ± 0.73	70.72 ± 15.57*
Cinnamon oil	28.93 ± 7.21***^#^	2.63	7.78 ± 0.56	81.08 ± 2.09***
Clove oil	21.64 ± 8.00*	1.97	7.52 ± 0.53	71.24 ± 15.85*

a Cumulative amount of permeated ibuprofen at 48 h/added ibuprofen*100%.

* *p* < 0.05,

** *p* < 0.01,

*** *p* < 0.001 vs. the vehicle group,

# *p* < 0.05 vs. the Azone group.

### ATR-FTIR studies

**Figure 3.**
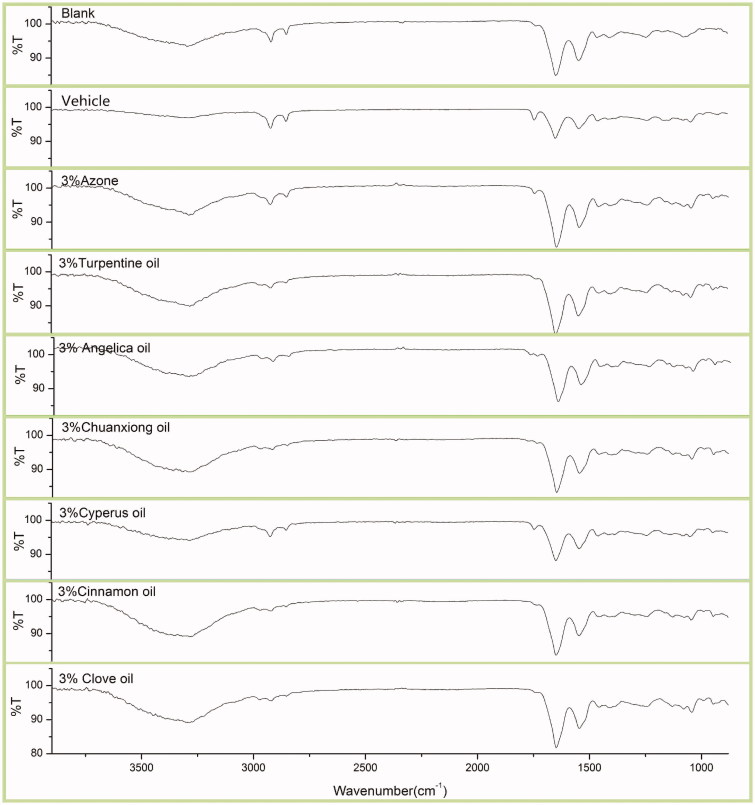
ATR**-**FTIR absorption spectra of rat skin treated with 3% w/v EOs.

According to literature reports, mechanisms of action of EOs are mainly based on changing the structure of the SC barrier and interaction with intercellular SC lipids to increase diffusivity of drugs (Herman & Herman 2014). ATR-FTIR has already been proved to be a promising tool to study the spatial organization of SC lipids (Laugel et al. 2005). The ATR-FTIR spectra of rat SC treated with different EOs are displayed in Figure 3. The changes of peak position and peak area are presented in Table 3 and Table 4, respectively.

**Table 3.** Peak positions of SC lipids after treated with 3%EOs (*n* = 3).

	Peak position of lipid (cm^−1^)		The shift of peak position (Δcm^−1^)^a^		
PE	Asymmetric C-H stretching	Symmetric C-H stretching	Asymmetric C-H stretching	Symmetric C-H stretching	Total of the decrease
Blank	2917.05 ± 1.15	2849.58 ± 0.31	0	0	0
Vehicle	2919.83 ± 0.76	2850.14 ± 2.28	2.83	0.56	3.39
Azone	2925.17 ± 2.03	2851.23 ± 0.52	8.12	1.65	9.77
Turpentine oil	2923.23 ± 1.28	2850.33 ± 1.24	6.18	0.75	6.93
*Angelica* oil	2921.72 ± 2.72	2849.80 ± 1.99	4.67	0.22	4.89
Chuanxiong oil	2923.94 ± 2.76	2851.28 ± 0.20	6.89	1.70	8.59
*Cyperus* oil	2921.98 ± 1.02	2851.04 ± 0.24	4.93	1.46	6.39
Cinnamon oil	2922.86 ± 2.76	2851.58 ± 1.48	5.81	2.00	7.81
Clove oil	2920.75 ± 0.24	2850.87 ± 1.51	3.70	1.29	4.99

a Shift in peak position compared to the untreated skin treated (Blank).

**Table 4.** Peak areas of SC lipids after treated with different EOs (*n* = 3).

	Peak area		The decrease of peak area^a^		
PE	Asymmetric C-H stretching	Symmetric C-H stretching	Asymmetric C-H stretching	Symmetric C-H stretching	Total of the decrease
Blank	84.12 ± 1.94	33.47 ± 3.14	0	0	0
Vehicle	81.77 ± 0.97	36.99 ± 0.62	−2.35	3.52	1.17
Azone	61.39 ± 5.14	25.38 ± 9.64	−22.73	−8.09	−30.82
Turpentine oil	33.72 ± 4.65	15.77 ± 7.00	−50.40	−17.70	−68.10
*Angelica* oil	40.61 ± 0.56	15.11 ± 2.00	−43.51	−18.36	−80.23
Chuanxiong oil	38.58 ± 7.98	12.96 ± 4.12	−45.54	−20.51	−66.05
*Cyperus* oil	44.41 ± 0.77	14.74 ± 3.34	−39.71	−18.73	−58.44
Cinnamon oil	28.60 ± 3.81	8.89 ± 0.97	−55.52	−24.58	−80.1
Clove oil	33.09 ± 12.33	10.33 ± 0.44	−51.03	−23.14	−74.07

a Decrease in peak area = peak area of EO treatment–peak area of blank.

**Figure 4.**
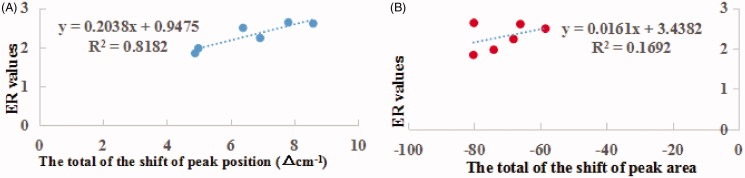
The relationship between ER values and the total of the shift of peak position (A) or the total decrease of peak area (B) after EOs treatment.

Figure 4 depicts the relationship between the ER values (Table 2) of EOs and the total of the shift of peak position (Table 3) or the total of the decrease of peak area (Table 4). Linear relationship (*r^2^* = 0.8182, *r* = 0.9045) was found between ER values and the total of the shift of peak position. However, for the decrease of the peak area, no linear relationship was found with ER values.

### Cell viability assay

**Figure 5.**
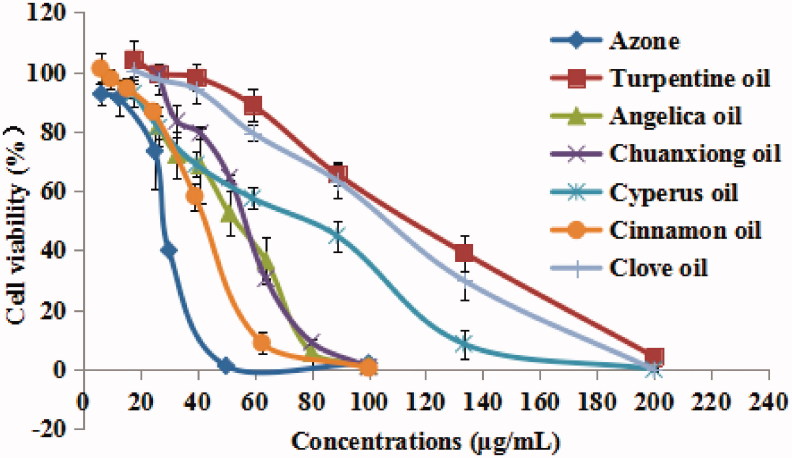
Effect of EOs with different concentrations on HaCaT keratinocyte cell viability *in vitro* (*n* = 6).

Most PEs have been shown to produce skin irritation or toxicity in spite of their satisfactory performance in enhancing skin permeation of drugs. Therefore, few of them have been approved for clinical use. The toxicities of six EOs and azone against skin cells were measured and compared using MTT assay. The results of HaCaT cells treated with different concentrations of EOs are shown in Figure 5. It was found that all examined EOs induced dose-dependent cytotoxicity. The calculated IC_50_ values of turpentine, *Angelica*, chuanxiong, *Cyperus*, cinnamon, clove oils and azone were determined to be 118.48, 53.26, 58.14, 81.80, 42.62, 109.03, and 21.13 μg/mL, respectively. The decreasing trend of skin cell toxicity was azone > cinnamon oil> *Angelica* oil > chuanxiong oil > *Cyperus* oil > clove oil > turpentine oil. The results suggested that the natural EOs possessed relatively lower skin irritation potential due to their natural origin (Fox et al. 2011). Furthermore, it was not found that skin cell toxicity was directly related with skin penetration enhancement effect.

## Discussion

The best model for skin permeation evaluation is the human skin, avoiding any species extrapolation. However, restricted access to human material, limited availability and high costs are the obstacle to use native human skin. Consequently, a number of animal skin models have been used to perform *in vitro* skin permeation studies. Due to its barrier properties comparable to human skin as well as better availability, rat skin is widely applied for the permeation studies to replace human skin (Takeuchi et al. 2011). Hence in the present study, the rat abdominal skin was used after removing the hair.

It was difficult for hydrophobic EOs to dissolve in water. Therefore, although some organic solvents possessed penetration enhancing capacity, they were usually applied as the composition of the vehicle to dissolve both EO and the drug. To investigate the penetration enhancing potential of tulsi and turpentine oils on percutaneous permeation through rat abdominal skin of flurbiprofen, a binary solvent mixture of PG:IPA (3:7, v/v) was used to dissolve both flurbiprofen and EOs (Charoo et al. 2008). In the present study, the binary solvent of PG: IPA (3:7, v/v) was also used as vehicle to dissolve both ibuprofen and EOs. And the penetration enhancement effect or the skin cell toxicity of EOs were determined and compared with blank vehicle to offset the effect of vehicle. Hydrophobic vehicle might delay the partition of lipophilic ibuprofen form vehicle to SC, resulting in the prolonged lag time. However, using hydrogel as vehicle, the lag time of ibuprofen was found to be almost zero in the *in vitro* skin permeation tests (Khan et al. 2011; Jiang et al. 2016) and the T_max_ of ibuprofen in rat plasma was determined to be 7.5 h in the *in vivo* pharmacokinetic studies (Jiang et al. 2016). It should be noted that the penetration enhancement effect of EOs may be different in different vehicle systems due to the differences in physico-chemical properties of these solvents and their interactions with SC.

Since they are volatile compounds, the boiling points of terpenes play a major role in their penetration enhancement efficacy (Narishetty & Panchagnula 2004). Mechanisms of action of the terpenes, derived from EOs, are mainly based on changing the structure of the SC barrier and interaction with intercellular SC lipids to increase diffusivity of drugs (Herman & Herman 2014). The low boiling points of terpenes indicate the weak cohesiveness or self-association of the molecules and therefore they may be more freely available to interact with lipids of SC and alter the barrier property (Das et al. 2006; Jain et al. 2008). Consequently, although with the highest SC/vehicle partition coefficient, the lowest volatility of clove oil led to the low penetration enhancement effectiveness. However, in this study, it was also found that too high volatility of EO, i.e. turpentine oil, was harmful to its penetration enhancement effect. After application to the surface of skin, the highly volatile components were quickly evaporated before sufficient interaction with SC lipids could happen. In summary, EOs with moderate volatility (0.759 ∼ 1.67 mg/h/cm^2^), i.e. chuanxiong, *Cyperus*, and cinnamon oils, possessed satisfactory penetration enhancement effectiveness.

In this study, to elucidate the effect of EOs on the intercellular lipid in the SC, ATR-FTIR stretching peaks near 2850 cm^−1^ (CH symmetric stretching absorbance frequency peak) and 2920 cm^−1^ (CH asymmetric stretching absorbance frequency peak) were measured to provide specific information about the interior composition of SC lipids before and after the application of 3% w/v EO to rat skin. These bands can be attributed to the methylene groups of the SC lipid alkyl chains. It has been demonstrated that PEs which cause a shift to a higher CH_2_ stretching frequency improve drug permeation (Casiraghi et al. 2010; Furuishi et al. 2013). And the decrease of peak area for both CH symmetric and asymmetric stretching absorbance frequency peak caused by PEs also indicated a disruption of the intercellular lipid bilayers (Vaddi et al. 2002; Casiraghi et al. 2010; Lan et al. 2014a).

The shift to a higher frequency occurs when methylene groups of the SC lipid alkyl chains change from *trans* to *gauche* conformation, suggesting that the SC lipid is disturbed. The magnitude of blue shift in the peak frequency of the asymmetric and symmetric stretching vibration absorbance is correlated with an increased number of *gauche* conformers in the lipid acyl chain (Furuishi et al. 2013). The areas of these two peaks are in proportional to the amount of the lipids present in SC. Therefore, any extraction of the lipids by EOs results in a decrease of peak area (Vaddi et al. 2002).

Figure 4 depicts the relationship between the ER values (Table 2) of EOs and the total of the shift of peak position (Table 3) or the total of the decrease of peak area (Table 4). A linear relationship (*r^2^* = 0.8182, *r* = 0.9045) was found between ER values and the total of the shift of peak position. However, for the decrease of the peak area, no linear relationship was found with ER values. The results indicated that the EOs promoted the skin permeation of ibuprofen mainly by disturbing rather than extracting the SC lipids. In addition, contribution to the partitioning of lipophilic ibuprofen into SC was also the minor mechanism of skin penetration enhancement of EOs.

It is important to find an optimum balance between the safety and potency of EOs. In conclusion, among investigated EOs, chuanxiong oil has been demonstrated to be the best PE for the percutaneous absorption of ibuprofen. By GC-MS analysis, its main components were determined to be ligustilide, 2-propyl-phenol and butylidenephthalide with relative contents of 41.00, 10.01 and 7.08%, respectively. The dose of 1 g/kg chuanxiong oil (approximately 2020 times of clinical dose used) had no observable side effect on guinea pig skin in the skin sensitization test (Zhang et al. 2012). As PE for flurbiprofen, chuanxiong oil showed significant penetration enhancement efficiency. Different concentrations (1%, 3%, 5%, 10% and 15%) of EO were compared and 3% chuanxiong oil appeared to obtain the highest flux in the permeation studies (Zhang et al. 2006). In addition, by percutaneous administration, chuanxiong oil suppresses hypertrophic scarring in the rabbit ear model and is a probably an effective cure for human hypertrophic scarring (Wu et al. 2011).

## Conclusions

The obtained data confirm that EOs possess higher penetration enhancement effectiveness and lower skin cell toxicity compared with azone, the classical PE. And chuanxiong oil has been demonstrated to be the best PE for the percutaneous absorption of ibuprofen. Turpentine, chuanxiong, *Cyperus*, cinnamon, and clove oils led to the significant increase of the SC/vehicle partition coefficients of ibuprofen, which indicated that these EOs might promote its partitioning into SC. However, the major mechanism of action for the investigated EOs has been proved to be the disintegration of the highly ordered intercellular lipid structure in SC. The results of ATR-FTIR revealed a linear relationship (*r^2^* = 0.8182, *r* = 0.9045) between ER values and the total of the shift of peak position, indicating that the EOs promoted the skin permeation of ibuprofen mainly by disturbing rather than extracting the SC lipids. Furthermore, it was also found that EOs with moderate volatility might have satisfactory penetration enhancement effectiveness.
